# Development and Phytochemical Characterization of High Polyphenol Red Lettuce with Anti-Diabetic Properties

**DOI:** 10.1371/journal.pone.0091571

**Published:** 2014-03-17

**Authors:** Diana M. Cheng, Natalia Pogrebnyak, Peter Kuhn, Christian G. Krueger, William D. Johnson, Ilya Raskin

**Affiliations:** 1 Department of Plant Biology and Pathology, Rutgers, The State University of New Jersey, New Brunswick, New Jersey, United States of America; 2 Complete Phytochemical Solutions, Cambridge, Wisconsin, United States of America; 3 Pennington Biomedical Research Center, Baton Rouge, Louisiana, United States of America; Kobe University, Japan

## Abstract

Polyphenol-rich Rutgers Scarlet Lettuce (RSL) (*Lactuca sativa* L.) was developed through somaclonal variation and selection in tissue culture. RSL may contain among the highest reported contents of polyphenols and antioxidants in the category of common fruits and vegetables (95.6 mg/g dry weight and 8.7 mg/g fresh weight gallic acid equivalents and 2721 µmol/g dry weight and 223 µmol/g fresh weight Trolox equivalents). Three main compounds accumulate at particularly high levels in RSL: chlorogenic acid, up to 27.6 mg/g dry weight, cyanidin malonyl-glucoside, up to 20.5 mg/g dry weight, and quercetin malonyl-glucoside, up to 35.7 mg/g dry weight. Major polyphenolic constituents of RSL have been associated with health promotion as well as anti-diabetic and/or anti-inflammatory activities. Daily oral administration of RSL (100 or 300 mg/kg) for up to eight days acutely reduced hyperglycemia and improved insulin sensitivity in high fat diet-induced obese hyperglycemic mice compared to vehicle (water) control. Data presented here support possible use of RSL as a functional food for the dietary management of diabetes.

## Introduction

Chronic metabolic diseases, including diabetes, cardiovascular disease, and obesity, are a growing concern worldwide. Modifications in diet and exercise can have a significant impact in preventing and amending metabolic diseases [1]. Epidemiological data suggest that diets high in fruits and vegetables decrease the risk of chronic non-communicable diseases [2]–[5]. Growing research on plant polyphenolic compounds, including green tea catechins, wild blueberry anthocyanins, cranberry proanthocyanidins, cocoa flavan-3-ols, coffee hydroxycinnamates, and many others suggest that they are important for conferring benefits to human health [6]–[8].

Anthocyanins, in particular, are of interest due to anti-oxidant, anti-inflammatory and anti-diabetic effects [9]–[11]. Dietary supplementation with an anthocyanin-rich extract from bilberry and blueberry improved hyperglycemia and insulin sensitivity in type 2 diabetic mice [12], [13]. Additionally, diabetic mice given a diet supplemented with the anthocyanin cyanidin 3-glucoside for eight weeks reduced hepatic oxidative damage and prevented the development of hepatic steatosis compared to mice on a control diet [11]. In another study, five weeks of dietary supplementation of cyanidin 3-glucoside to high fat diet-fed and *db/db* mice lowered fasting blood glucose, improved insulin sensitivity, alleviated hepatic steatosis, and reduced inflammatory cytokines in adipose tissue compared to non-supplemented controls [14]. Polyphenols from blueberry and grape sorbed, stabilized, and concentrated to soy flour demonstrated anti-diabetic effects, including lowering blood glucose and reducing weight gain in high fat diet-induced obese mice [15], [16]. In a clinical study, obese and insulin-resistant subjects consuming a beverage containing 45 g of whole blueberry powder (1.3 g of anthocyanins) showed significant improvement in insulin sensitivity compared to placebo control [17]. Another randomized, double-blind, crossover intervention study showed significant and rapid improvements (1 h post consumption) in flow-mediated vascular dilation in patients receiving flavonoid-rich blueberry powder containing 319 to 1791 mg of total blueberry polyphenols [18].

Our aim was to develop and characterize common foods with a particularly high content of bioactive polyphenols using non-transgenic approaches. In this study, we focused on red leaf lettuce as a dietary source of beneficial polyphenols. Lettuce is an important crop widely consumed throughout the world; thus small nutritional enhancements can have a significant health impact. According to the United Nations Food and Agriculture Association, worldwide production of lettuce (and chicory) was over 24 million tons with a gross production of over $14 billion in 2012 [19]. While lettuce is one of the most commonly consumed vegetables, nevertheless, it is not often associated with health-promoting properties, although several reports suggest that health benefits of lettuce may be attributed to polyphenols as well as vitamins, carotenoids, and fiber [20].

Here we report the successful use of non-transgenic tissue culture technologies to induce somaclonal variations and to select and generate lettuce cultivars with very high polyphenolic content, named Rutgers Scarlet Lettuce (RSL). As anthocyanins are polyphenolic pigments responsible for red, blue, and purple coloration in fruits and vegetables, visual clonal selection in tissue culture was used to detect variants with high polyphenolic content. We also provide biochemical characterization of RSL polyphenols and report on the acute glucose lowering and insulin sensitizing activity of the RSL leaf powder in a mouse model of diet-induced obesity and diabetes.

## Materials and Methods

### Materials

Murashige and Skoog (MS) basal salts, growth regulators and agar were purchased (Phytotechnology Labs, Shawnee Mission, KS). Trolox® [(±)-6-Hydroxy-2,5,7,8-tetramethylchromane-2-carboxylic acid, 97%], fluorescein sodium salt, 2,2′-Azobis(2-methylpropionamidine)dihydrochloride (AAPH), phosphate buffered saline (PBS) 10× concentrate, Folin-Ciocalteu phenol reagent, caffeic acid, cyanidin 3-galactoside, and organic solvents were purchased from Sigma (St. Louis, MO). Quercetin 3-rhamnoside was purchased from HWI Analytik GMBH (Ruelzheim, Germany).

### Development of Rutgers Scarlet Lettuce in Tissue Culture

#### Seed Germination and Micropropagation

Seeds of seven lettuce cultivars (Red Romaine Rhazes, Red Romaine Annapolis, Red Lollo Natividad, Red Grand Rapids Firecracker, Dark Red Lollo Rossa, Red Lollo Antago and Red Grand Rapids Blackhawk) were surface sterilized by immersion in 70% ethanol for 1 min, followed by a 1.2% sodium hypochlorite solution for 15 min and rinsed three times with sterilized distilled water. Seeds were placed in Magenta GA-7 boxes with 40 mL of MS germination medium (Table 1) [21]. All media pH was adjusted to 5.7 and solidified with 0.7% agar and autoclaved at 121°C, 103 kPa for 20 min. Seeds were germinated at 23°C with a 16 h-light/8 h-dark photoperiod. Continuous micropropagation of cultivars was maintained by transferring 1 cm stem segments with axillary buds onto propagation medium (Table 1) and subcultured every 5–6 weeks. For root induction, well developed shoots were excised and transferred to MS basal medium (Table 1).

**Table 1:** Rutgers Scarlet Lettuce tissue culture media.

Media	MS Basal Salts	Sucrose (%)	Growth Regulators
Basal	1×	3.0	-
Germination	1/2×	1.0	-
Propagation	1×	3.0	0.5 mg/L BAP
Callus induction	1×	3.0	0.3 mg/L BAP, 2.0 mg/L NAA, 0.2 mg/L 2,4-D
Callus propagation	1×	3.0	0.5 mg/L 2,4-D

Media pH was adjusted to 5.7 and solidified with 0.7% agar.

MS: Murashige and Skoog basal salts.

BAP: 6-benzylaminopurine.

NAA: naphthaleneacetic acid.

2,4-D: 2,4-dichlorophenoxyacetic acid.

#### Plant Regeneration and Callus Induction

To develop an efficient regeneration system, 5-day-old cotyledons and leaf segments (0.7×0.7 cm) were placed on MS media with various concentrations of growth regulators (Table 2). Explants were cultivated for 6 weeks and evaluated for shoot regeneration efficiency.

**Table 2:** Rutgers Scarlet Lettuce regeneration media.

Growth Regulators, mg/L	R1	R2	R3	R4	R5	R6	R7	R8	R9	R10	R11
6-Benzylaminopurine	1.0	-	-	2.0	1.0	1.0	1.0	0.5	1.0	1.0	2.0
Zeatin	-	1.0	-	-	1.0	0.5	0.5	-	1.0	0.5	-
Kinetin	-	-	1.0	-	-	-	-	-	-	-	-
Naphthaleneacetic acid	0.1	0.1	0.1	0.1	0.1	0.1	0.2	-	-	0.1	0.1
2,4-Dichlorophenoxyacetic acid	-	-	-	-	-	-	-	-	-	0.1	0.2

Base medium: Murashige and Skoog basal medium with 3% sucrose and 0.7% agar.

For callus induction, cotyledons or leaf segments were placed in Petri plates on callus induction medium (Table 1). Plates were incubated under darkness at 23°C for 4–6 weeks. Well developed calli were selected and transferred to callus propagation medium (Table 1) to obtain friable callus and subcultured every 3–4 weeks. Callus tissues were cultivated for 3–4 months and then transferred to regeneration media to determine optimal medium for shoot development (Table 2).

#### Selection and Production of Anthocyanin-Rich Regenerants

Anthocyanin production and accumulation was used as a visual selection marker during shoot regeneration and propagation in tissue culture conditions. Regenerants from different types of explants showed a wide range of color including green, red, and dark red/purple especially under UV supplementation (light intensity 80 µE m^−2^ s^−1^; Philips F40T12/DX E6 ALTO, Philips Lighting Co, Mountain Top, PA). The darkest red/purple regenerants/segments were excised and placed again on regeneration media (Table 2). Several plantlets, exhibiting and maintaining a dark red phenotype, were produced after multiple rounds of regeneration and selection and transplanted to soil containers. Seeds were collected from self-pollinated transplants and germinated in growth chamber conditions to produce F1 plants. For phytochemical and functional analyses, outer leaves were sampled from 2–3 month-old plants of the darkest red F1 plants. Lettuce plants were maintained in growth chambers (19°C day, 16°C night, 16 h- light/8 h-dark photoperiod, 65% relative humidity, and 225 µE m^−2^ s^−1^ light intensity provided by high intensity white fluorescent lamps (Philips F96T12/CW/VHO). F1 plants with the highest levels of anthocyanins and total polyphenols were self-pollinated and F2 seeds were produced and germinated in growth chambers. Leaves from 2–3 month-old F2 plants collected from the darkest red plants were used for phytochemical and functional analyses. Lines with the highest content of anthocyanins were self-pollinated for production of F3 and F4 generations. Seeds were deposited with the American Type Culture Collection (ATCC) with the following designations: NAR-S-13 PTA-120680, NBR-S-16 PTA-120681, NFR-S-4 PTA-120682.

### Phytochemical Analyses

#### Extraction

Fresh leaves were frozen at −80°C prior to lyophilization. Dried leaves were ground to a powder with a mortar and pestle. Leaf extracts were prepared as described [22] with minor modifications. Briefly, 0.5 g of dried material was transferred to a 50 mL tube protected from light and 15 mL of extraction solvent (methanol/water/acetic acid; 85∶14.5∶0.5) was added. Samples were vortexed for 30 s, sonicated for 5 min, vortexed for 30 s, then incubated at room temperature for 10 min. Samples were again vortexed for 5 s then centrifuged at 1699 rcf for 5 min. The supernatant was decanted and the extraction process was repeated two more times. The supernatants were pooled and filtered through 0.45 µm PTFE filters (VWR, Radnor, PA) prior to analyses. Extracts were stored at −20°C.

#### Total Polyphenols

Total phenolic content was measured by a modified Folin-Ciocalteu method [23], [24]. Briefly, Folin-Ciocalteu phenol reagent was combined with 50% methanol (1∶1). The Folin-Ciocalteu solution was added to 200 µL of standard dilutions or sample dilutions and incubated at room temperature for 10 min. Next, 300 µL of 2 M sodium carbonate was added and samples were incubated in a 40°C water bath for 20 min. Samples were cooled on ice and centrifuged for 30 s at 7000 rcf. The supernatant was transferred to a 96-well plate and absorbance was measured at 760 nm in triplicate on a Synergy HT Multi-Detection Microplate Reader (Bio-Tek, Winooski, VT). Results were expressed as gallic acid equivalents ± standard error (SE) on a dry weight (DW) or fresh weight (FW) basis.

#### Anthocyanins

Total monomeric anthocyanin content was determined according to the AOAC pH differential method [25]. Samples were diluted as needed and pH 1 (25 mM potassium chloride) or pH 4.5 (0.4 M sodium acetate) buffers were added to each sample. Absorbances of the pH 1 and pH 4.5 solutions were measured at 510 nm and 700 nm in triplicate in a plate reader. Anthocyanin content was expressed as cyanidin 3-glucoside equivalents ± SE.

#### Oxygen Radical Absorption Capacity (ORAC)

ORAC was measured as described [26] with slight modifications. All reagents and sample dilutions were made in 75 mM PBS, pH 7.4. Stock solutions of fluorescein 1 mM and Trolox 2.5 mM were prepared and single use aliquots were stored at −20°C. Water was pipette into the outer wells of a 96-well plate. Fluorescein was diluted to 6 nM from the stock solution and 150 µL of was added to each well. Next, 25 µL of Trolox standard (0–50 µM) or samples were added to each well in triplicate. The plate was pre-incubated at 37°C for 30 min. A fresh solution of AAPH was prepared and 25 µL added to each well after pre-incubation to a final concentration of 16 mM. Fluorescence readings were recorded every minute for 75 min on the Synergy microplate reader (485 nm, 20 nm bandpass, excitation filter and a 528 nm, 20 nm bandpass, emission filter). A regression line was generated by plotting the concentration of Trolox by net area under the curve (AUC). Results were expressed as µmol Trolox equivalents (TE) ± SE.

Additionally, lyophilized leaves were sent to Brunswick laboratories (Southborough, MA) for external validation of antioxidant capacity by ORAC-H [27], [28].

#### High Performance Liquid Chromatography (HPLC) Analysis of Hydroxycinnamic Acids, Anthocyanins and Flavonols

Separation and analysis of RSL phenolics were performed by analytical HPLC. Methanolic extracts were injected (100 µl) onto a C-18 column (Waters ODS-2, 25 cm×0.45 cm; Milford, MA). The solvents for elution were 0.1% trifluoroacetic acid in water (solvent A) and methanol (solvent B). A step gradient program was developed that optimized the separation of phenolics. The program was 90% A to 72% A over 10 min under a linear gradient, isocratic for 20 min, 72% A to 45% A over the next 20 min under linear gradient, then 100% solvent B over the next 5 min with a linear gradient. Waters Empower software was used to generate a 3-dimensional (3D) data set (absorbance, retention time, and wavelength) for the analyte and calibration standards. Six point (1, 5, 10, 25, 50 and 100 ppm) calibration curves were developed to allow for quantification (peak area) of hydroxycinnamic acids, anthocyanins and flavonols.

#### Matrix-Assisted Laser Desorption/Ionization Time-of-Flight Time-of-Flight Mass Spectrometry (MALDI/TOF/TOF/MS)

Mass spectra were collected on a Bruker ULTRAFLEX®III MALDI/TOF/TOF Mass Spectrometer (Billerica, MA, USA) equipped with delayed extraction and a SmartBeam® laser. All analyses were performed in positive reflectron mode. Spectra were the sum of 8–10 different locations in each well, accumulating a total of 400–500 shots to minimize intra-well variability and avoid heterogeneous co-crystallization spots. Threshold laser power was used to achieve optimal isotope patterns. The matrix was 2,5-dihydroxybenzoic acid at a concentration of 50.0 mg/mL in ethanol. FlexControl and FlexAnalysis (Bruker Daltonik GmbH, Bremen, Germany, version 3.0) were used for data acquisition and data processing, respectively. Mass (version 3.9.0) was used for spectra analysis.

#### Liquid Chromatography Mass Spectrometry (LCMS)

Methanolic extract samples were separated and analyzed with a Shimadzu LCMS-2010A high performance liquid chromatography/electrospray ionization/single quadrupole mass spectrometer. The LCMS is equipped with two pumps (LC-10ADvp), controller (SCL-10Avp), auto-injector (SIL-10ADvp), column oven (CTO-10ACvp), photodiode array detector (SPD-M10Avp), and a single quadrupole analyzer. A Wide Pore C5, 5 µm, 2.1×150 mm column (Supelco, Cat# 568402-u) was used. Elution gradient conditions were as follows: Solvent A: water (0.1% formic acid) and solvent B: acetonitrile (0.1% formic acid) with a flow rate of 0.2 mL/min. The gradient used was: initially 95% A and 5% B; linear gradient to 75% A and 25% B over 10 min; linear gradient to 5% A and 95% B over 2 min, followed by isocratic elution at 5% A and 95% B for 3 min; returning to starting conditions by linear gradient 95% A and 5% B over 1 min. A 13 min equilibration interval was run between injections.

### *In Vivo* Experiments

#### Ethics Statement

All animal experiments were performed according to *Guide for the Care and Use of Laboratory Animals* of the National Research Council. Protocols were approved by the Internal Animal Care and Use Committee at Rutgers, The State University of New Jersey (# 04-023).

#### Animal Care

Five week-old male C57Bl/6J mice were purchased from Jackson Labs (Bar Harbor, ME) and housed in cages (five mice to a cage) at 25°C with a 12 h light-dark cycle. Mice were given a one week acclimatization period and fed a chow diet (Purina, No. 5015) with *ad libitum* access to food and water. For a diet induced obese (DIO) mouse model, mice were fed a very high fat diet (VHFD) containing 60% kcal fat (D12492, Research Diets, New Brunswick, NJ) for at least 12 weeks, whereby mice become obese, hyperglycemic, and insulin resistant [29]. All mice were introduced to intragastric feeding prior to treatment. During this time, a gavage administration of 0.2 mL of double distilled water was performed daily for 2–3 days prior to the scheduled experiments. Weekly food intake per cage and body weights was recorded throughout the study.

#### Acute Fasting Blood Glucose (FBG) Test and Insulin Tolerance Test

Acute FBG tests and insulin tolerance test were performed on DIO mice fed VHFD for 14 weeks. Similarly, DIO mice were gavaged daily with RSL (NFR-S-4) at a dose of 100 or 300 mg/kg suspended in water or vehicle as a negative control (*n* = 8 per treatment group). A Metformin group (300 mg/kg) was included as positive control (*n* = 4). On day 6 of treatment, acute effects of RSL were determined by fasting mice for 4 h and measuring FBG before and 6 h after treatment. On day 8 of treatment, mice were given insulin tolerance tests. Mice were fasted for 4 h prior to insulin i.p. injection (0.7 U/kg) and blood glucose was recorded every 20 or 30 min from the tail vein using an AlphaTrak handheld glucometer (Abbott Labs Inc., Abbott Park, IL).

#### Oral Glucose Tolerance Test

Oral glucose tolerance tests were performed on another set of DIO mice given a VHDF for 17 weeks. A preliminary oral glucose tolerance test was given and mice were divided to balanced glucose response groups. RSL leaves were lyophilized and ground to a fine powder with a mortar and pestle. DIO mice were orally gavaged daily with RSL (NFR-S-4) at a dose of 100 or 300 mg/kg suspended in water or with vehicle (water) control (*n* = 10 per treatment group). A Metformin group (300 mg/kg) was included as positive control (*n* = 5). After 7 days of treatment, mice were fasted for 6 h and given an oral glucose challenge (2 g/kg) and blood glucose was recorded every 30 min.

### Statistical Analysis

Data are presented as mean ± SE. Statistical significance of differential effects of RSL, Metformin and vehicle control were determined using a general linear model analysis of variance. Effect of treatment of DIO mice with lyophilized and powdered RSL leaves was assessed on day 6 of on-study treatment by comparing change in FBG after 6 h of acute treatment on day 6 of daily treatment. Analysis of variance was performed to obtain a global test of differences among low and high concentrations of RSL, Metformin, and control followed by *post-hoc t*-tests to assess significance of RSL linear dose-response trend (RSL doses 0, 100 and 300 mg/kg), low dose RSL versus control, high dose RSL versus control and high dose RSL versus Metformin. Longitudinal data from insulin tolerance tests and oral glucose tolerance tests were analyzed by applying a mixed effects model for repeated measures across 120 min. Dunnett's multiple comparisons *post-hoc* tests were used to determine significant differences between treatments for area under the curve data sets. A global significance level was set at α = 0.05. All statistical procedures were performed with SAS 9.3 software (Cary, IN).

## Results

### Development of Somaclonal Variants

To produce somaclonal variants with the highest degree of visible anthocyanin coloration (dark red/purple pigment), several methods for inducing variation and selective pressure were used independently or in combination, including multiple regeneration cycles, regeneration from cotyledons, leaves, and callus, as well as selection for dark red/purple color, which was most visible in plants grown under high intensity light with some UV present in the spectrum. Shoot regeneration from cotyledons and young leaves, with average regeneration rates of 70% and 50%, respectively, was the most efficient on regeneration medium R7 (Table 2).

**Figure 1:**
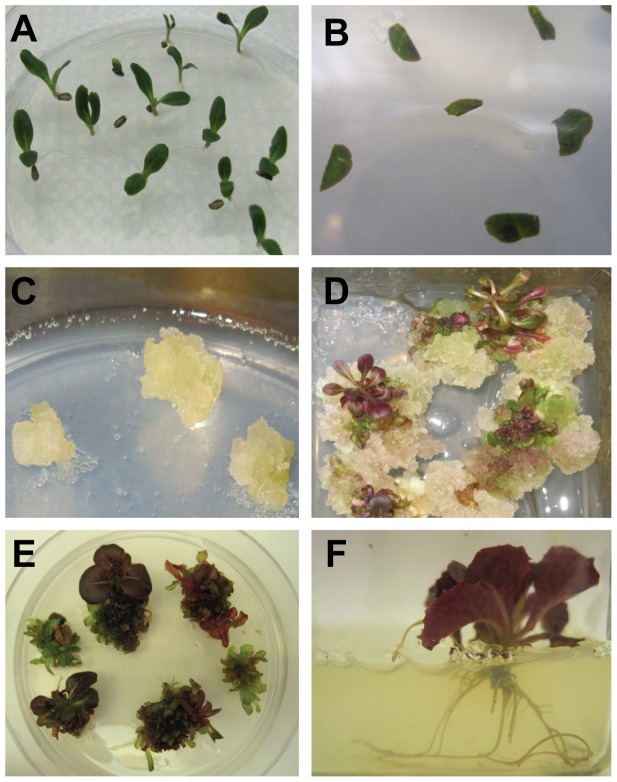
Development of Rutgers Scarlet Lettuce through plant tissue culture. **A** Seed germination. **B** Cotyledon segments for callus induction. **C** Callus production. **D** Shoot regeneration from callus. **E** Shoot induction and selection for anthocyanin-rich phenotype. **F** Root induction from shoots.

**Figure 2:**
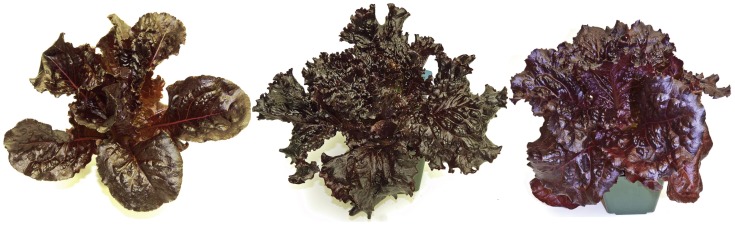
Rutgers Scarlet Lettuce lines NAR-S-13, NBR-S-16, NFR-S-4 (left to right).

Cotyledons or young leaves were also used as explants for callus production (Fig. 1 A and B). A high percent of callus, 51–62%, was produced on callus induction medium (Table 1). Initial calli were transferred to callus propagation medium (Table 1) to produce fast-growing friable callus (Fig. 1C). After 3–4 months of cultivation, callus tissues were transferred to regeneration media (Table 2; Fig. 1D). Regeneration medium R9, with 31–36% regeneration, was most efficient for shoot regeneration from callus. Shoots exhibited a wide range of color from green to red/purple (Fig. 1E). The darkest red/purple shoots/segments were cut into pieces and re-cultured on regeneration medium R7. The process of shoot selection and regeneration was repeated. After several rounds of regeneration and selection, 15 dark-red lines were produced from three commercial cultivars of red lettuce: Red Grand Rapids Blackhawk (NBR lines), Red Grand Rapids Firecracker (NFR lines) and Red Romaine Annapolis (NAR lines). These lines were transferred to MS medium for root induction, showing initial root development after 5–6 days and well-formed roots after 3 weeks (Fig. 1F). Plantlets (F0) were transplanted to soil containers and grown to maturity in a greenhouse. Seeds were produced from the darkest F0 red lettuce lines after self-pollination and germinated in the growth chamber to produce F1 plants. The darkest red F1 plants were visually selected, phytochemically analyzed and termed Rutgers Scarlet Lettuce (RSL). Three RSL lines (NFR-S-4, NBR-S-16 and NAR-S-13) with the highest anthocyanin content were self pollinated again to produce F2 lines for phytochemical and functional analyses reported in this manuscript (Fig. 2). F2 plants of all three RSL cultivars showed little color variation.

### Phytochemical Analysis and Oxygen Radical Absorption Capacity

Phytochemical analysis revealed that RSL cultivars (NFR-S-4 and NBR-S-16) grown under 19°C day, 16°C night, 16 h-light/8 h-dark photoperiod, 65% relative humidity, and 225 µE m^−2^ s^−1^ light intensity provided by high intensity white fluorescent lamps, contained high amounts of total polyphenols (95–96 mg/g DW of leaves) and anthocyanins (22–26 mg/g DW of leaves) (Table 3). Additionally, RSL cultivars had high antioxidant capacity, 2445–2721 µmol TE/g DW (Table 3). On average, RSL contained 91.1% water and on a FW basis. RSL accumulated 8.7 mg/g total polyphenols (NBR-S-16), 2.1 mg/g anthocyanins (NFR-S-4), and 223 µmol TE/g (NFR-S-4) (Table 3).

**Table 3:** Phytochemical content of Rutgers Scarlet Lettuce cultivars.

	Total Polyphenols (mg/g)		Anthocyanins (mg/g)		ORAC (µmol/g)		ORAC* (µmol/g)	
	DW	FW	DW	FW	DW	FW	DW	FW
NFR-S-4	94.9±2.5	7.8±0.3	25.8±2.1	2.1±0.2	2721±286	223±23	2287	183
NBR-S-16	95.6±5.3	8.7±0.3	22.2±2.3	2.0±0.1	2445±127	222±24	1934	155

DW: Dry weight basis; FW: Fresh weight basis.

Total polyphenols are reported as gallic acid equivalents ± SE.

Anthocyanins are reported as cyanidin 3-glucoside equivalents ± SE.

ORAC (oxygen radical absorbance capacity) values are reported as Trolox equivalents ± SE.

*Determined by Brunswick Labs (Southborough, MA).

Independent validation of the ORAC values obtained by our team for NFR-S-4 and NBR-S-16 were performed by Brunswick Labs (Southborough, MA). Analysis performed in both locations showed similar results (Table 3).

### Quantification of Hydroxycinnamic Acids, Anthocyanins and Flavonols by HPLC

**Figure 3:**
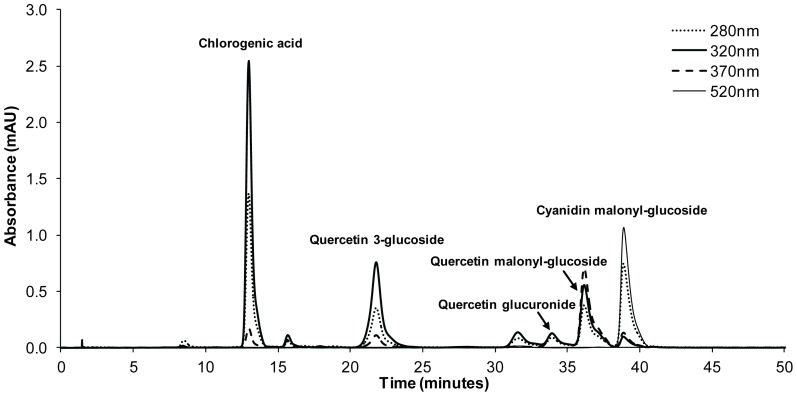
Representative HPLC chromatogram of Rutgers Scarlet Lettuce (line NFR-S-4) extract.

Analysis of RSL phenolics (cultivars NAR-S-13, NBR-S-16, NFR-S-4) were performed by analytical HPLC. Commercially available standards, representative of the compounds found naturally in red leaf lettuce, were chosen as calibration standards for quantification of phenolics by HPLC: caffeic acid for hydroxycinnamic acids (320 nm), quercetin 3-rhamnoside for flavonols (370 nm) and cyanidin 3-galactoside (520 nm) for anthocyanins. The composite chromatograph served as a phytochemical fingerprint for RSL that provides a qualitative and quantitative chemical profile. A representative chromatogram of the acidified methanolic extract of NFR-S-4 is shown in Fig. 3. Each peak of the analyte 3D HPLC data set was inspected and maximum absorbance was determined. The HPLC chromatogram of analytes contained three main classes of polyphenols: 1) hydroxycinnamic acids, 2) flavonols, and 3) anthocyanins, reported earlier for the red leaf lettuce (Table 4). Compounds were tentatively identified by MALDI/TOF/TOF/MS.

**Table 4:** Phytochemical composition of Rutgers Scarlet Lettuce.

UV-Max (nm)	m/z [M+H]^+^	Compound	NFR-S-4 NBR-S-16 NAR-S-13
			mg/g dry weight		
354	355	Chlorogenic acid	23.9	27.6	26.8
333	ND	Hydroxycinnamic acid	0.7	0.7	0.4
326	465	Quercetin 3-glucoside	13.1	9.9	8.7
327	ND	Hydroxycinnamic acid	2.7	4.0	2.7
516	ND	Anthocyanin	0.2	0.3	0.3
349	479	Quercetin 3-glucuronide	6.9	6.7	4.5
333	551	Quercetin 3-malonyl-glucoside	35.7	31.9	30.9
516	535	Cyanidin 3-malonyl-glucoside	20.5	17.0	18.3

Hydroxycinnamic acids have a characteristic maximum absorbance between 310–320 nm. A chromatogram at 320 nm was extracted from the analyte 3D HPLC data set. Analyte peaks that had a 310–320 nm maximum absorbance were observed to elute from the column between 8–34 min. By calculating the area only under these peaks and applying the calibration curve for caffeic acid [y = 3,089,958x+994,428 (R^2^ = 1.00)] the amount of hydroxycinnamic acid present in the analyte was determined (Table 4).

Flavonols have a characteristic maximum absorbance between 350–370 nm. A chromatogram at 370 nm was extracted from the analyte 3D HPLC data set. The analyte peaks that had this characteristic were observed to elute from the column between 28–38 min. By calculating the area only under these peaks and applying the calibration curve for quercetin 3-rhamnoside [y = 1,060,244 x−8,952 (R^2^ = 1.00)] the amount of flavonols present in analyte was determined (Table 4). It should be noted that while maximum absorbance of flavonols is between 350–370 nm, they also have absorbance characteristics at 320 nm.

Anthocyanins have a characteristic maximum absorbance around 520 nm. A chromatogram at 520 nm was extracted from the analyte 3D HPLC data set. The analyte peaks that had this characteristic were observed to elute from the column between 32–40 min. By calculating the area only under these peaks and applying the calibration curve for cyanidin 3-galactoside [y = 2,382,163x+479,868 (R^2^ = 1.00)] the amount of anthocyanins present in analyte was determined (Table 4). It should be noted that while anthocyanin maximum absorbance is around 520 nm they also have absorbance characteristics at 320 nm and at 370 nm.

### RSL Acutely Reduces Hyperglycemia and Improves Insulin Sensitivity

**Figure 4:**
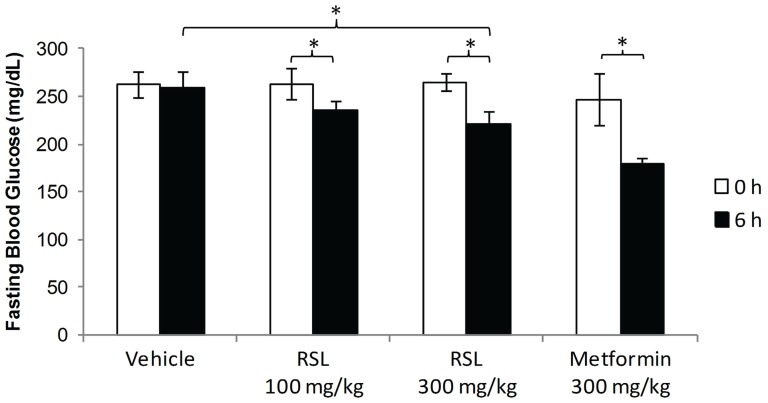
Acute fasting blood glucose-lowering effects of Rutgers Scarlet Lettuce (RSL, line NFR-S-4). Diet-induced obese mice given RSL (100 and 300 mg/kg), water (vehicle), or Metformin (*n* = 8, except Metformin, *n* = 4). After 6 d of treatment, mice were fasted for 4 h and blood glucose was measured immediately prior to and 6 h after gavage. Data are presented as the mean ± SE. Significant differences were analyzed comparing the change in fasting blood glucose from 0 h and 6 h after treatment, comparing differences in the change in FBG with 100 or 300 mg/kg RSL treatment vs control, and comparing differences in the change in FBG with 100 or 300 mg/kg RSL treatment vs Metformin (* *p*<0.05).

**Figure 5:**
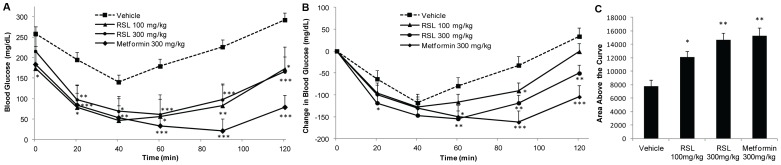
Insulin sensitizing effects of Rutgers Scarlet Lettuce (RSL, line NFR-S-4). Diet-induced obese mice were given daily oral administrations of RSL, water (vehicle), or Metformin (*n* = 8, except Metformin group, *n* = 4). On day 8 of treatment, mice were fasted for 4 h, given 0.7 U insulin i.p. injection and blood glucose was recorded at indicated intervals. **A** Mean blood glucose (mg/dL) across time. **B** Mean change in blood glucose across time. **C** Area above the curve of the change in blood glucose across time. Data are presented as the mean ± SE. Significant differences vs vehicle control (* *p*<0.05, ** *p*<0.01, *** *p*<0.001).

Treatment of DIO mice with lyophilized and powdered RSL (NFR-S-4) leaves acutely lowered FBG and improved insulin response as measured by insulin tolerance test. An acute FBG test was given on day 6 of treatment, resulting in decreased FBG after 6 h of active treatment (100 mg/kg RSL, 300 mg/kg RSL, or Metformin) compared to 0 h (Fig. 4). Specifically, FBG decreased significantly in each of the three active treatment groups (all *p*<0.05), but not in the control. There was a significant decreasing linear dose-response trend in FBG across mice receiving respective concentrations of 0, 100, and 300 mg/kg RSL. Decrease in FBG was significantly greater in mice receiving 300 mg/kg RSL versus control (*p*<0.05) but not in mice receiving 100 mg/kg RSL versus control or in mice receiving 300 mg/kg RSL versus Metformin. Insulin tolerance tests were performed on day 8 of treatment and significant dose dependent effects of RSL on mean blood glucose concentrations (Fig. 5A) and the change in blood glucose (Fig. 5B) over time compared to vehicle control was observed, indicating increased insulin sensitivity from RSL and Metformin treatments. Area above the curve analysis also illustrates a dose dependent increase in insulin sensitivity with RSL treatment compared to vehicle control (Fig. 5C). There were no significant differences in daily feed intake or changes in bodyweight compared to the vehicle control group.

**Figure 6:**
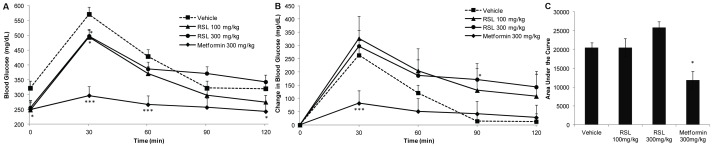
Oral glucose tolerance test after Rutgers Scarlet Lettuce (RSL, line NFR-S-4) treatments. DIO mice were given daily oral administrations of RSL, water (vehicle), or Metformin for 7 days (*n* = 10, except Metformin group, *n* = 6). Mice were fasted for 6 h prior to an oral glucose challenge and blood glucose was recorded at indicated intervals. **A** Mean blood glucose (mg/dL) across time. **B** Mean change in blood glucose across time. **C** Area under the curve of the change in blood glucose across time. Data are presented as the mean ± SE. Significant differences vs vehicle control (* *p*<0.05, ** *p*<0.01, *** *p*<0.001).

In another experiment, oral glucose tolerance tests were performed on DIO mice given RSL (NFR-S-4) for 7 days by oral gavage. Blood glucose levels of RSL treated groups were significantly lower than vehicle control only at 30 min post glucose challenge (Fig. 6A). Significant differences in the change in blood glucose over time were observed for RSL (300 mg/kg) 90 min post glucose challenge and for Metformin 30 min post glucose challenge (Fig. 6B). Area under the curve analysis of oral glucose tolerance tests only indicated a significant difference for Metformin compared to control and no significant difference with either RSL dose versus control (Fig. 6C).

## Discussion

Diets rich in fruits and vegetables are recommended for overall health and wellness and are associated with reduced risk of developing chronic metabolic diseases. As people are more aware of the impact of their dietary habits on health, there is growing consumer interest for functional foods that may provide benefits above basic nutrition. While yield and biotic and abiotic stress resistance still dominate crop improvement efforts in industry and academia, selection for phytochemical targets with health-promoting properties is slowly emerging [30]. To the best of our knowledge, this is the first report on the use of non-transgenic tissue culture technologies of selection and regeneration for enhancing health benefits of lettuce, which is one of the most commonly produced vegetables in the world. High polyphenol RSL was developed through visual selection for high levels of anthocyanin pigmentation in tissue culture. High levels of anthocyanins in RSL were associated with high levels of hydroxycinnamic acids and quercetin glucosides that together accounted for almost 10% of dried RSL leaves. These compounds have shown beneficial health effects *in vitro*, *in vivo* and in clinical studies. For example, insulin sensitivity in obese, non-diabetic, insulin-resistant patients [17] and vascular function in healthy men [18] were improved through dietary supplementation of blueberry powder. In addition, a recent large epidemiological study demonstrated that consumption of polyphenol-rich whole fruits reduced the risk of type 2 diabetes [31]. Dietary supplementation of quercetin improved inflammatory status, plasma insulin and lipids levels of obese rats [32], [33]. In humans, chlorogenic acid significantly lowered glucose and insulin concentrations 15 min after an oral glucose load compared with placebo in overweight men and acutely lowered blood pressure in healthy volunteers, suggesting mechanisms by which coffee may improve diabetes and cardiovascular diseases [34], [35]. Additionally, chlorogenic acid is an inhibitor of glucose-6-phosphatase enzyme system, a key regulator of hepatic glucose homeostasis [36].

Consistent with high polyphenol content, RSL has very high antioxidant capacity (Table 1) which, under optimum cultivation in high light, was 222 µmol TE/g FW, more than three times that of cultivated blueberry (47–62 µmol TE/g FW), a fruit marketed for its high antioxidant value [37]. Several publications present data regarding total polyphenol and anthocyanin content in different red lettuce cultivars growing under various conditions, including variable light intensity and temperature (Table 5). In comparison to these published data (compare Tables 3 and 5), RSL has the highest levels of total polyphenols (94.9 mg/g DW and 7.8 mg/g FW), the next highest cultivars being to Galactic (85.7 mg/g DW) and Lollo Rosso (6.5 mg/g FW). RSL has the greatest reported anthocyanin levels in red lettuce (2.1 mg/g FW), the next highest being Lollo Rosso cv. Revolution (0.99 mg/g FW).

**Table 5:** Phytochemical content of red lettuce cultivars.

Red Lettuce Cultivar	Total Polyphenols		Anthocyanins		Reference
	mg/g DW	mg/g FW	mg/g DW	mg/g FW	
Galactic	85.7				[58]
Galactic (abscisic acid treatment)	33.1		3.91		[40]
Galactic	28.2		1.74		[40]
Hongyil, Red Fire, Jinjuck, Dazzler, Fire, Seoul Red*	29.1			0.07	[59]
Red Oak Leaf	28.5				[56]
Red Oak Leaf (RZ)	23.1				[20]
Red Leaf *unspecified*	11.4		2.71		[55]
Lollo Rosso cv. Revolution		6.50		0.99	[38]
Lollo Rosso		5.71		0.46	[60]
Red Oak Leaf		3.22		0.26	[60]
Lollo Rosso		3.55			[42]
Red Oak Leaf		3.38			[42]
Lollo Rosso		1.76			[47]
Red Leaf *unspecified*		1.14			[37]
Cherokee		1.08			[61]
Red Sails		0.90			[43]
Valeria, OOC 1441, Impuls*			0.87		[62]
Hongyil				0.07	[63]

DW: Dry weight basis.

FW: Fresh weight basis.

* Values are the average of the cultivars listed.

Although the total polyphenol content is high in RSL, several factors can further enhance phytochemical accumulation in red lettuce. Factors include UV intensity [38], light quality (UV, B, green, or red) [39], treatment with exogenous abscisic acid [40], leaf position (inner/outer) [41], soil type [42], and growing conditions [43]. In addition to human health benefits, anthocyanins play important roles in plant health and protection from physiological and environmental stresses [44]. As such, environmental growth conditions can significantly affect the production and accumulation of plant secondary metabolites.

Based on published reports available to the authors, RSL has among the highest antioxidant capacity compared to other common fruits and vegetables on dry weight and fresh weight basis and per serving [37]. Samples were sent for independent evaluation of ORAC, and results were comparable to our values for the same sample batch of lettuce. For NFR-S-4, the TE values were 2218 µmol/g DW (our lab) and 2287 µmol/g DW (Brunswick Labs). For NBR-S-16, the TE values were 2233 µmol/g DW (our lab) and 1934 µmol/g DW (Brunswick Labs). The average ORAC values from our lab are presented in Table 3. Using serving sizes from Wu et al., (2004) [37], 68 g serving of RSL would provide approximately 15,100 µmol TE. RSL would be at the top of the list of foods categorized by hydrophilic ORAC per serving followed by small red kidney beans (13,727 µmol TE), lowbush blueberry (13,427 µmol TE), red kidney beans (13,259 µmol TE), pinto beans (11,864 µmol TE), cultivated blueberry (9,019 µmol TE) and cranberry (8,983 µmol TE) [37]. Red lettuce evaluated by Wu et al. [37], had 17.9 µmol TE/g FW. Others have reported enhanced ORAC values for red lettuce cultivars through exogenous abscisic acid application (Galactic 80.8 µmol TE/g DW) [40] and growth under UV-transparent plastic film (Lollo Rosso 850.5 µmol TE/g DW) [38]. While the benefit of antioxidants in disease prevention and human health is debated, diets rich in fruits and vegetables are accepted as recommended for human health maintenance and disease prevention.

Major polyphenolic constituents of RSL were similar to those reported in other cultivars of red lettuce, namely hydroxycinnamic acids and quercetin- and cyanidin-glycosides [40], [45]–[47]. These compounds are ubiquitous in plants and, as discussed, are associated with anti-diabetic, anti-inflammatory and anti-CVD activities [14], [48], [49] as well as with high antioxidant capacity [50], [51]. As these polyphenolic compounds are abundant in RSL, it is not surprising that RSL administration acutely reduced hyperglycemia and improved insulin sensitivity in DIO mice (Figs. 4 and 5). We hypothesize that chlorogenic acid has an important contribution to the anti-diabetic effects of RSL as it has been reported to reduce early glucose response in humans [34], demonstrated plasma bioavailability [35], and inhibited hepatic glucose production [36].

Although bioavailability of polyphenols is typically low, in the nanomolar range, levels in the gut lumen may reach the micromolar and millimolar range [52]. Polyphenols, including caffeoylquinic acids, flavonols, and anthocyanins, have been shown to inhibit the breakdown of starch and disaccharides (through inhibition of α-amylases and α-glucosidases) [53], [54], and/or inhibit sugar absorption via the transporters SGLT1 (sodium-glucose cotransporter 1) and GLUT2 (facilitated glucose transporter 2) [52]. The accumulated activities of multiple polyphenols in the intestinal lumen may lead to a reduction in postprandial blood glucose and help prevent the increase in fasting blood glucose over time. A future direction of research may focus on the capacity of RSL compounds to inhibit digestion and absorption of carbohydrates.

This is the first report that describes lettuce with anti-diabetic effects and provides biochemical characterization of its putative bioactives. We cannot exclude the possibility that public and private sources have developed other high polyphenol/high ORAC red lettuce varieties that still await biochemical and functional characterization. Previous studies on dietary supplementation with other red lettuce varieties have demonstrated improvement in lipid and antioxidant profiles in rodents [55], [56]. Additionally, lettuce contains vitamins C, E, luteolin and carotenoids that may contribute to health-promotion and cardiovascular disease mitigation [20], [57]. In conclusion, tissue culture selection was able to generate RSL, a red lettuce cultivar with one of the highest reported contents of polyphenols and antioxidants amongst common fruits and vegetables. Data presented here support possible use of RSL as a functional food for the dietary management of diabetes. Further pre-clinical and clinical validation of RSL will be required to justify such use.
